# Supplementary material for: The reliability and validity of a non-wearable indoor positioning system to assess mobility in older adults: A cross-sectional study
Source: PLoS One. 2025 Apr 25;20(4):e0307347. doi: 10.1371/journal.pone.0307347 (PMC12027111; doi:10.1371/journal.pone.0307347)

Results of Bland-Altman analysis

Chirp 126 vs Zeno	Bias (95% CI)	Upper limit of agreement (95% CI)	Lower limit of agreement (95% CI)
All experimental conditions	-0.009 (-0.018, -0.001)	0.039 (0.025, 0.054)	-0.058 (-0.073, -0.043)
Normal walk	-0.015 (-0.021, -0.008)	0.022 (0.011, 0.032)	-0.051 (-0.061, -0.039)
Obstacle walk	-0.002 (-0.012, 0.008)	0.050 (0.033, 0.067)	-0.053 (-0.071, -0.036)
Walk and talk	0.000 (-0.011, 0.012)	0.067 (0.047, 0.087)	-0.066 (-0.086, -0.046)
Narrow walk	-0.018 (-0.032, -0.003)	0.061 (0.035, 0.086)	-0.096 (-0.122, -0.071)
Fast walk	-0.026 (-0.039, -0.014)	0.044 (0.023, 0.066)	-0.097 (-0.119, -0.075)
**Chirp 201 vs Chirp 126**	**Bias (95% CI)**	**Upper limit of agreement (95% CI)**	**Lower limit of agreement (95% CI)**
All experimental conditions	-0.000 (-0.005, 0.004)	0.022 (0.015, 0.029)	-0.023 (-0.030, -0.016)
Normal walk	-0.000 (-0.006, 0.005)	0.031 (0.021, 0.041)	-0.032 (-0.041, -0.022)
Obstacle walk	0.006 (-0.001, 0.013)	0.040 (0.028, 0.052)	-0.028 (-0.040, -0.016)
Walk and talk	-0.000 (-0.004, 0.004)	0.021 (0.014, 0.027)	-0.021 (-0.028, -0.015)
Narrow walk	0.000 (-0.007, 0.007)	0.036 (0.024, 0.048)	-0.035 (-0.047, -0.023)
Fast walk	-0.005 (-0.015, 0.006)	0.053 (0.034, 0.071)	-0.062 (-0.080, -0.043)
**Chirp126 (Time 1 versus Time 3)**	**Bias (95% CI)**	**Upper limit of agreement (95% CI)**	**Lower limit of agreement (95% CI)**
All experimental conditions	0.055 (0.028, 0.081)	0.202 (0.156, 0.248)	-0.093 (-0.139, -0.047)
Normal walk	0.052 (0.012, 0.092)	0.271 (0.203, 0.340)	-0.167 (-0.235, -0.099)
Obstacle walk	0.054 (0.031, 0.078)	0.170 (0.130, 0.211)	-0.062 (-0.103, -0.022)
Walk and talk	0.085 (0.042, 0.127)	0.317 (0.243, 0.391)	-0.148 (-0.222, -0.074)
Narrow walk	0.084 (0.044, 0.124)	0.287 (0.218, 0.356)	-0.119 (-0.188, -0.049)

Fast walk	-0.025 (-0.063, 0.013)	0.180 (0.115, 0.245)	-0.230 (-0.296, -0.165)
**Chirp 201 Time 1 versus Time 3**	**Bias (95% CI)**	**Upper limit of agreement (95% CI)**	**Lower limit of agreement (95% CI)**
All experimental conditions	0.057 (0.028, 0.089)	0.212 (0.161, 0.263)	-0.097 (-0.148, -0.046)
Normal walk	0.051 (0.008, 0.093)	0.274 (0.200, 0.347)	-0.172 (-0.246, -0.099)
Obstacle walk	0.062 (0.035, 0.090)	0.193 (0.145, 0.241)	-0.068 (-0.116, -0.021)
Walk and talk	0.094 (0.051, 0.137)	0.320 (0.246, 0.395)	-0.133 (-0.207, -0.058)
Narrow walk	0.089 (0.049, 0.130)	0.287 (0.217, 0.358)	-0.108 (-0.179, -0.038)
Fast walk	-0.028 (-0.070, 0.013)	0.187 (0.114, 0.259)	-0.243 (-0.315, -0.171)

Plot chirp126 vs Zeno all experimental conditions

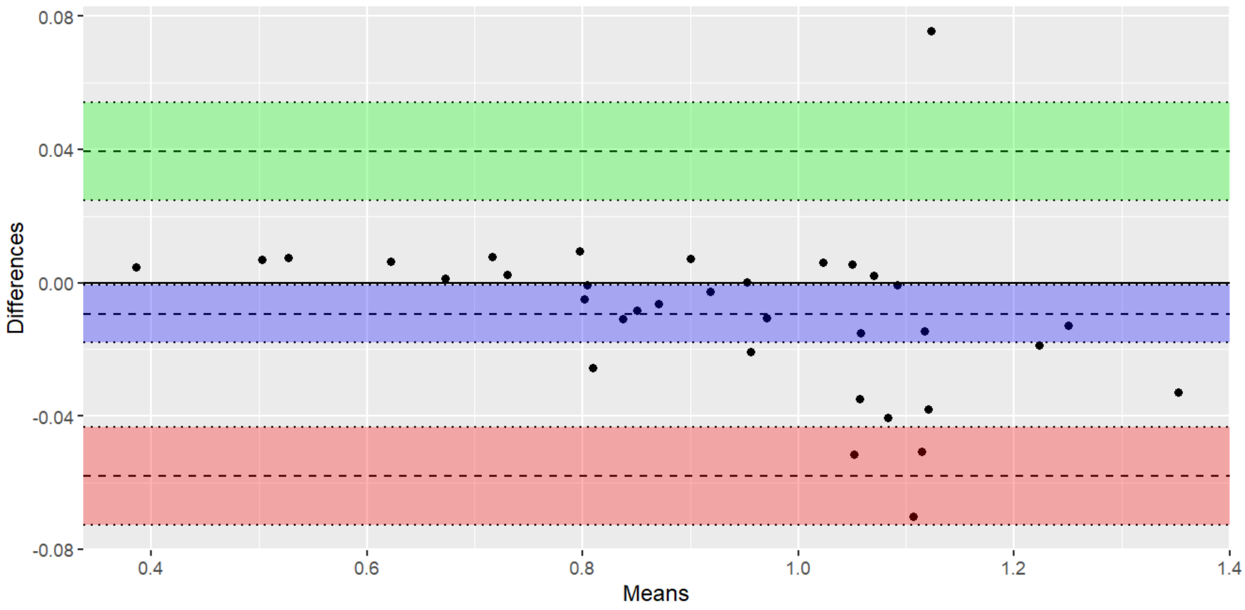

Plot chirp126 vs Zeno Normal Walk

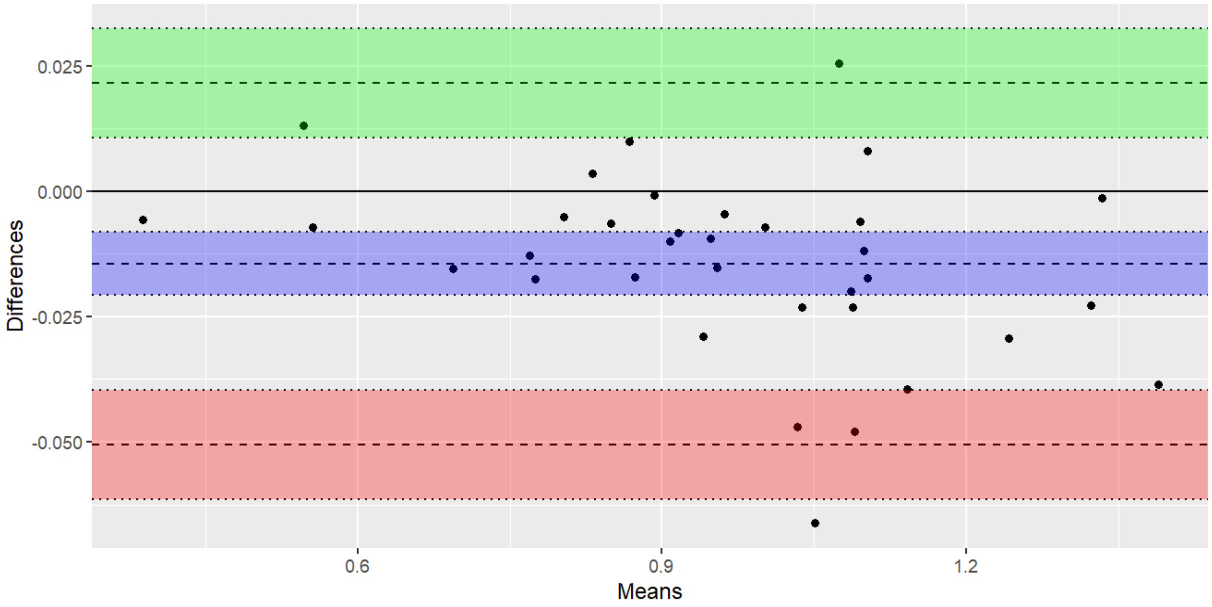

Plot chirp126 vs Zeno Obstacle Walk

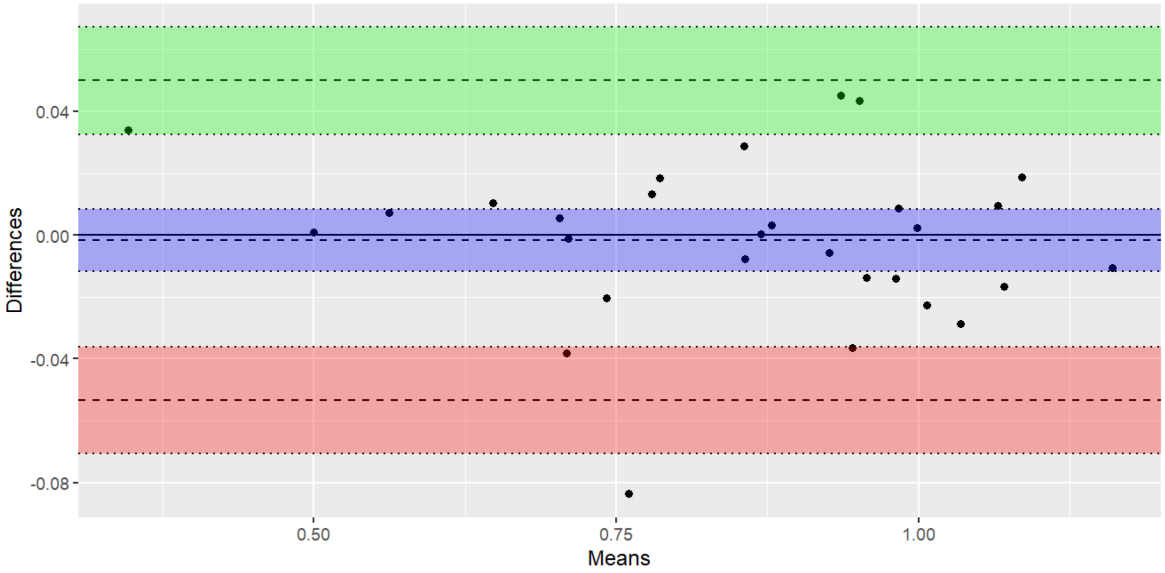

Plot chirp126 vs Zeno Walk and Talk

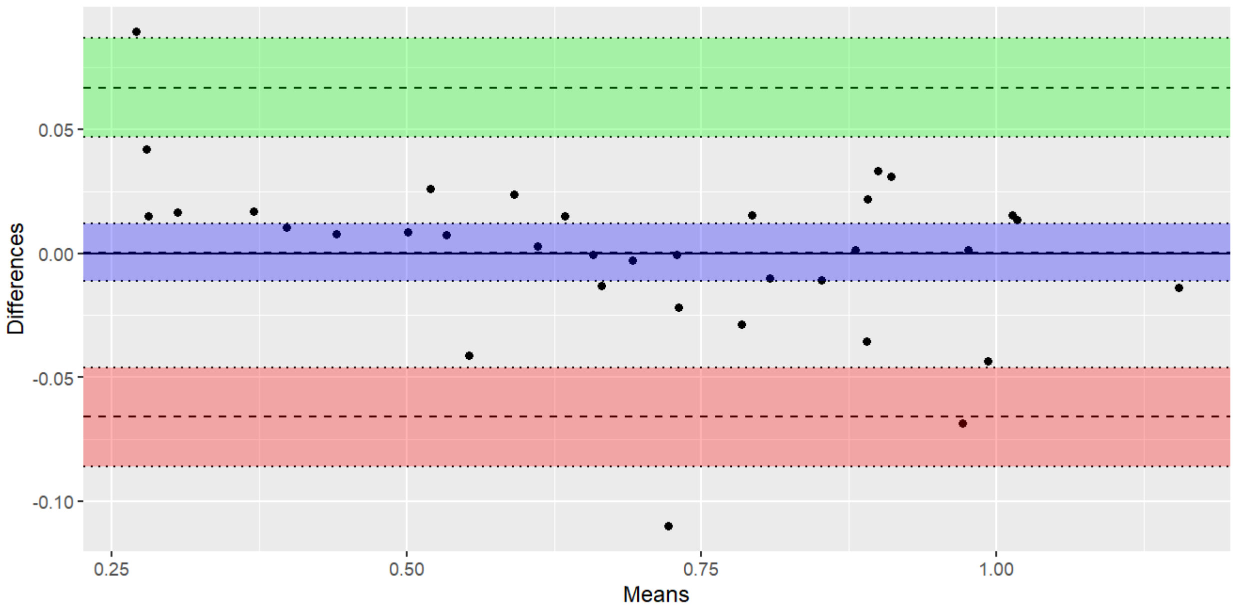

Plot chirp126 vs Zeno Narrow Walk

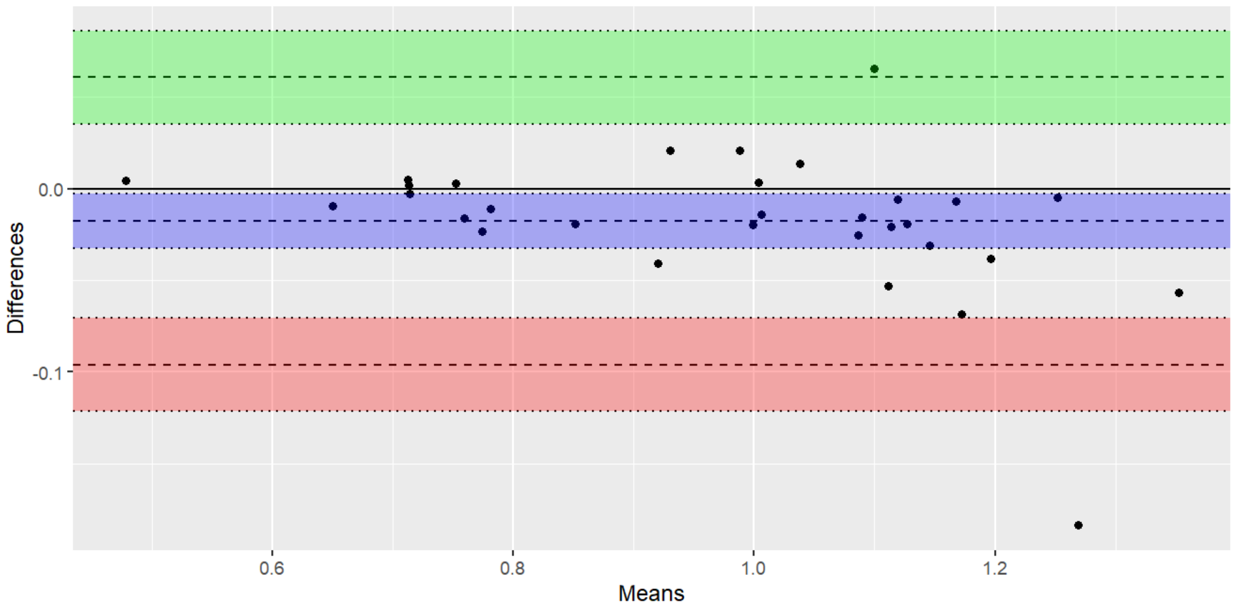

Plot chirp126 vs Zeno Fast Walk

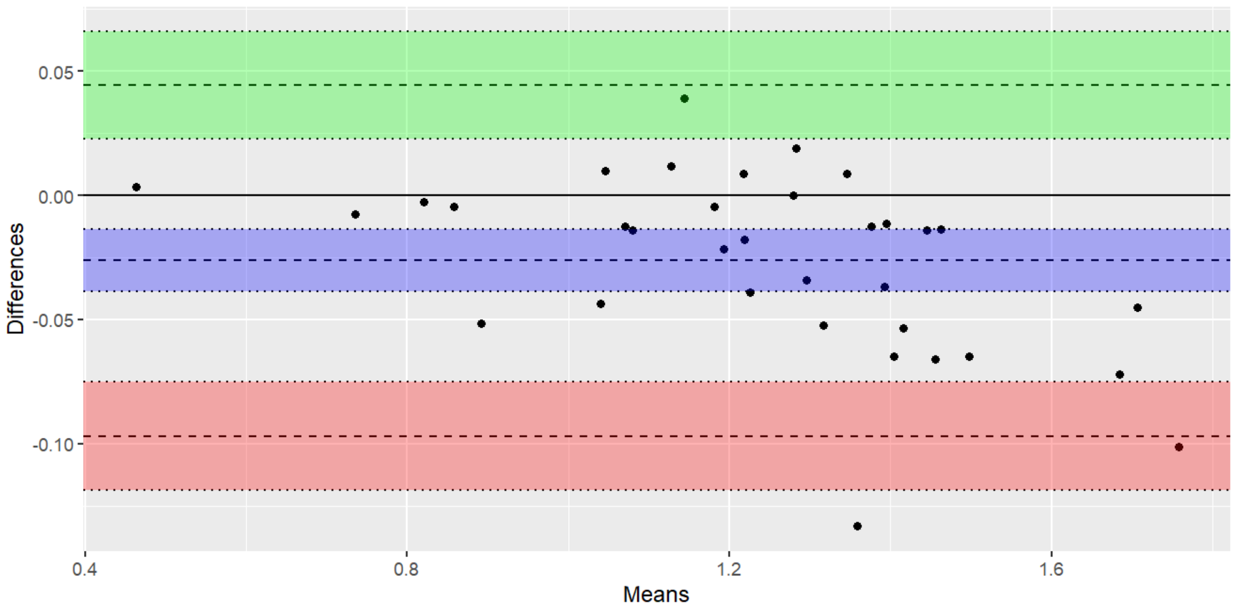

Plot chirp126 vs chirp201 all experimental conditions

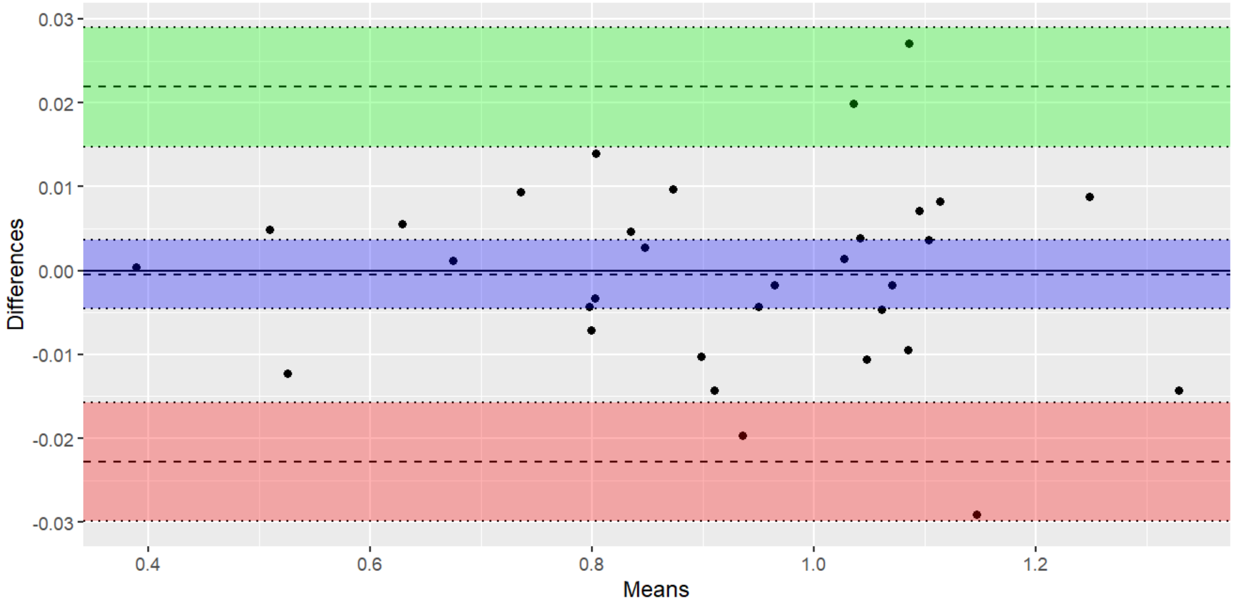

Plot chirp126 vs chirp201 Normal walk

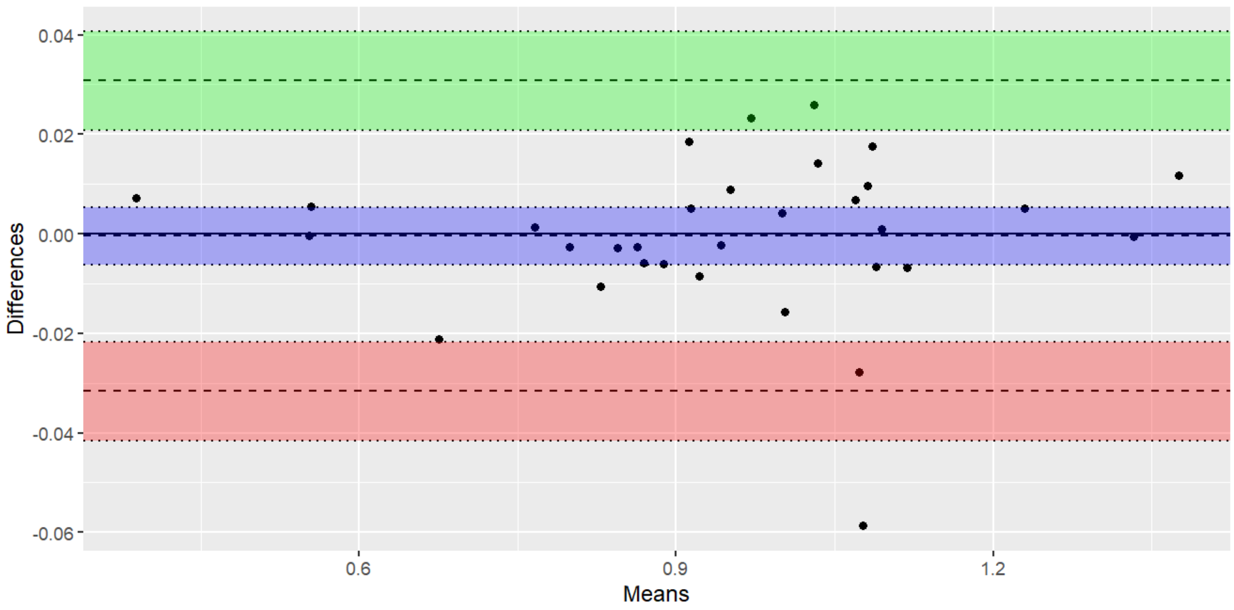

Plot chirp126 vs chirp201 obstacle walk

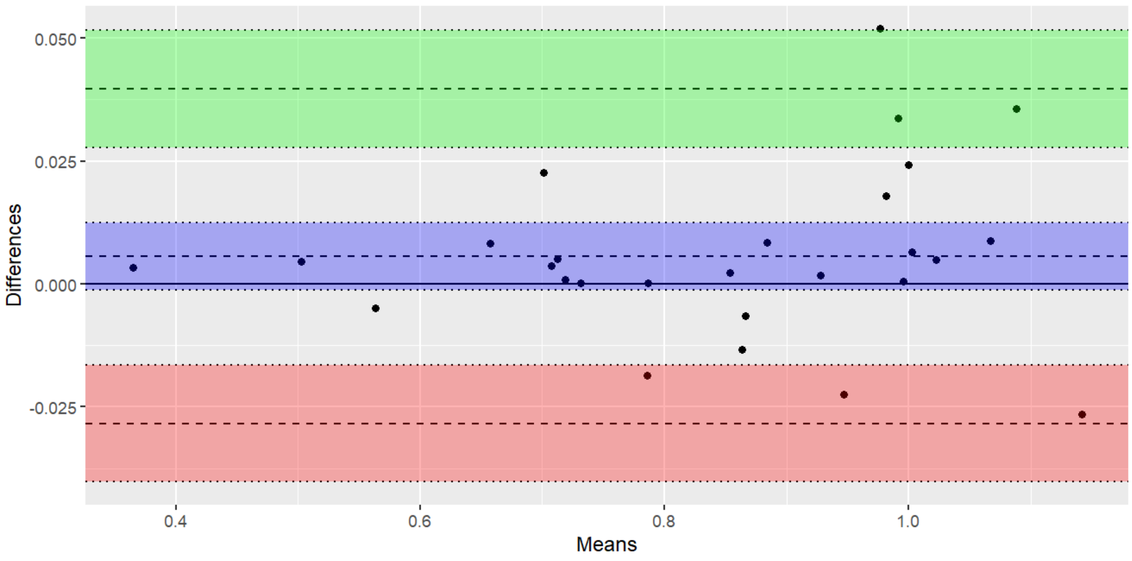

Plot chirp126 vs chirp201 walk and talk

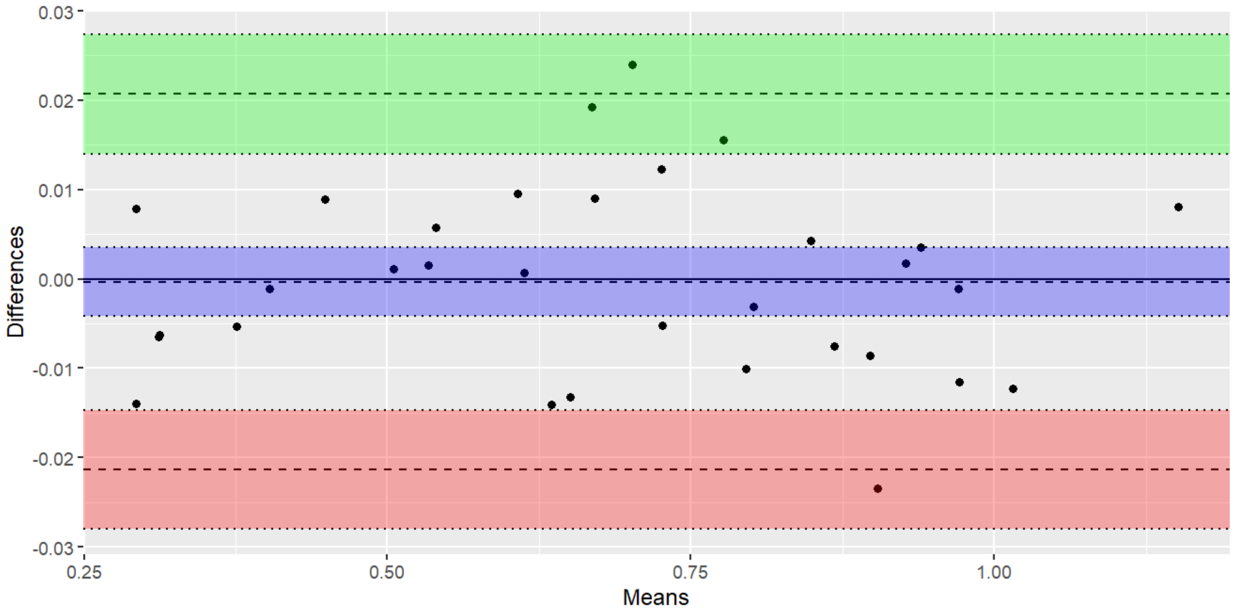

Plot chirp126 vs chirp201 narrow walk

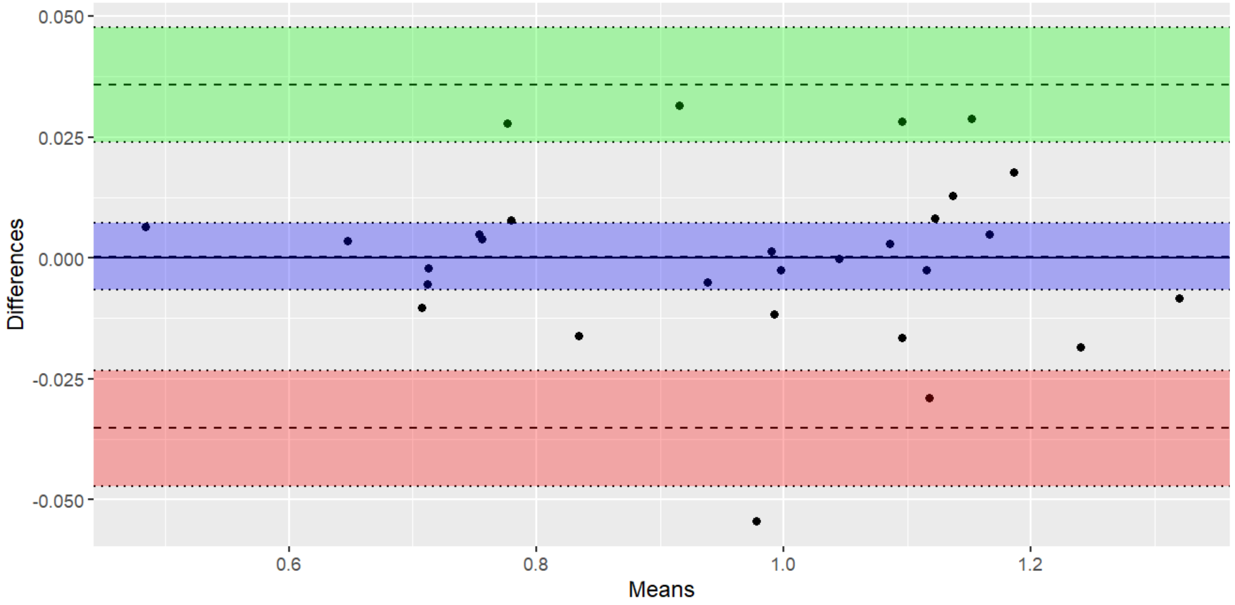

Plot chirp126 vs chirp201 fast walk

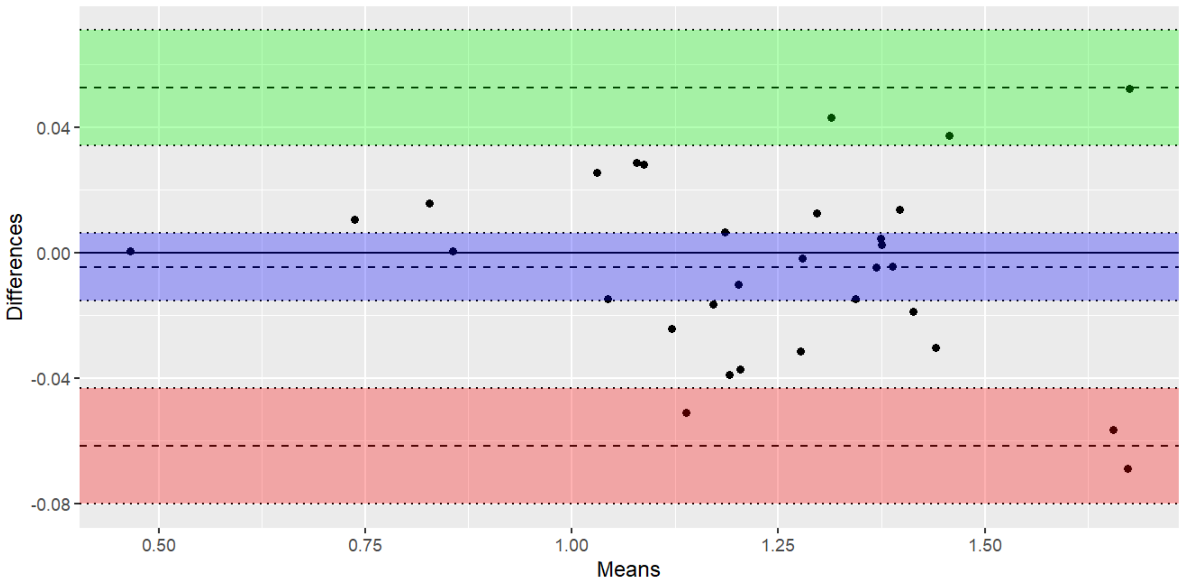

Chirp126 time 3 vs time 1 all experimental conditions

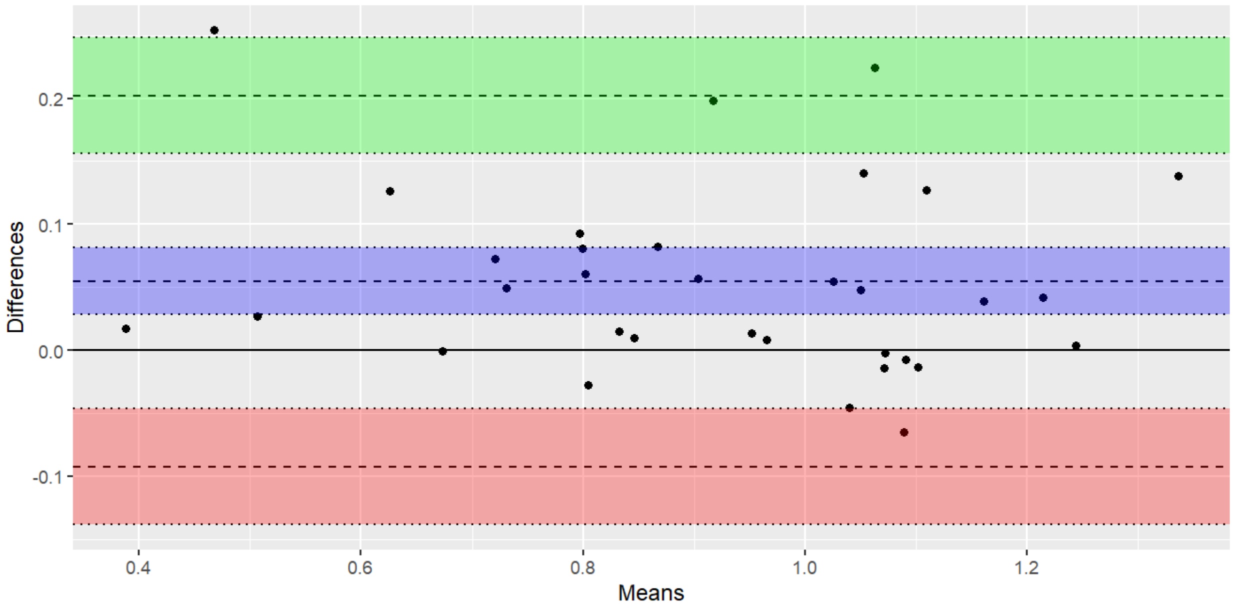

Chirp126 time 3 vs time 1 normal walk

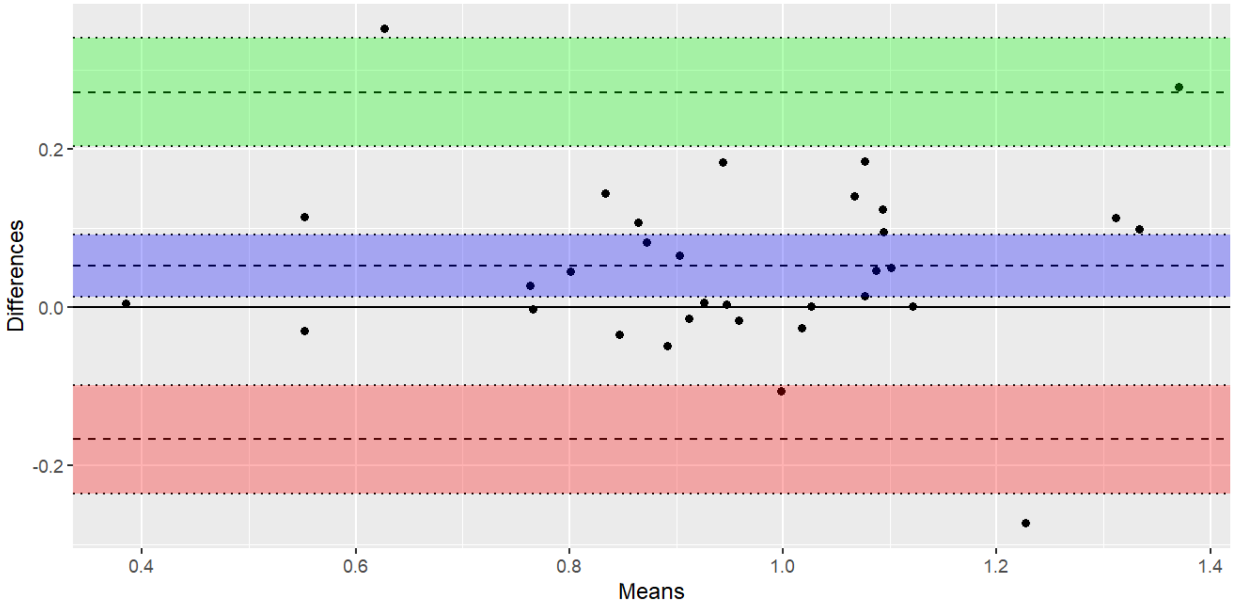

Chirp126 time 3 vs time 1 obstacle walk

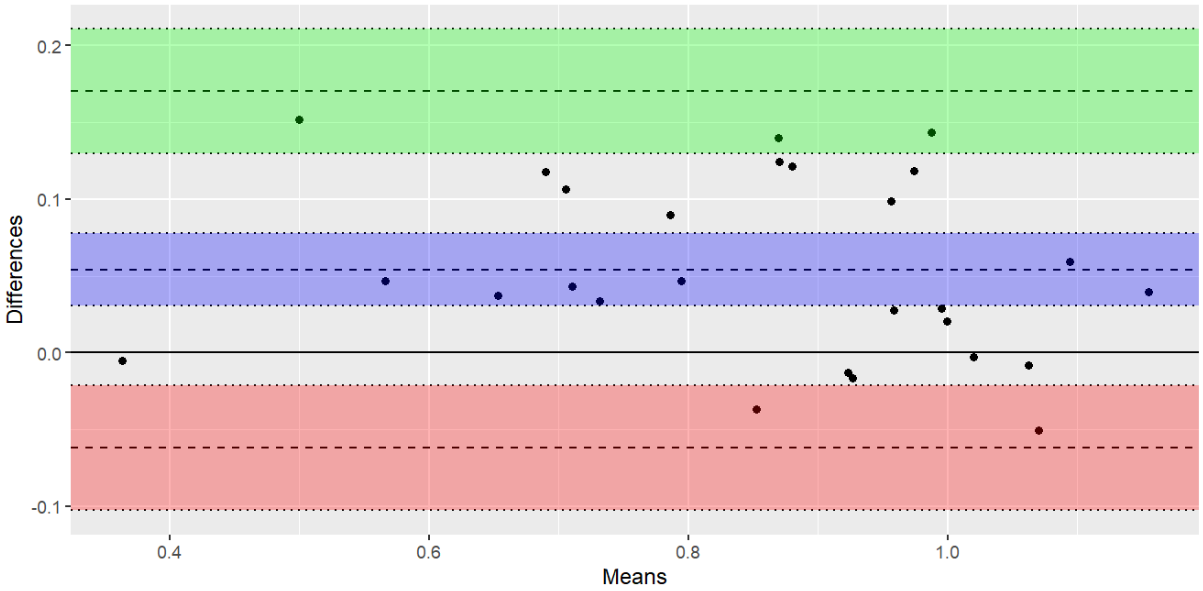

Chirp126 time 3 vs time 1 walk and talk

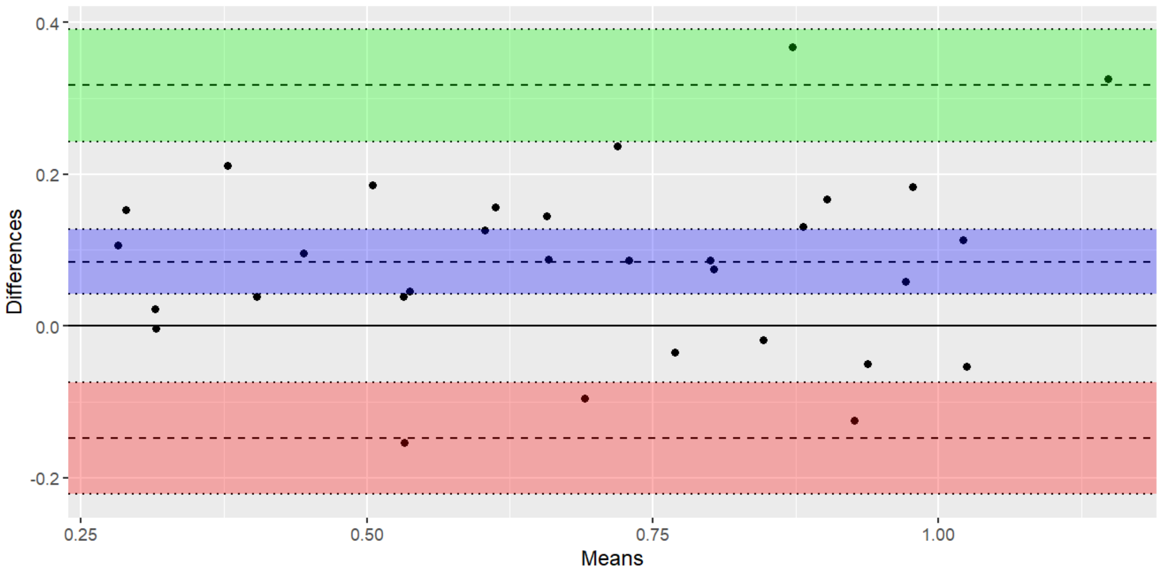

Chirp126 time 3 vs time 1 narrow walk

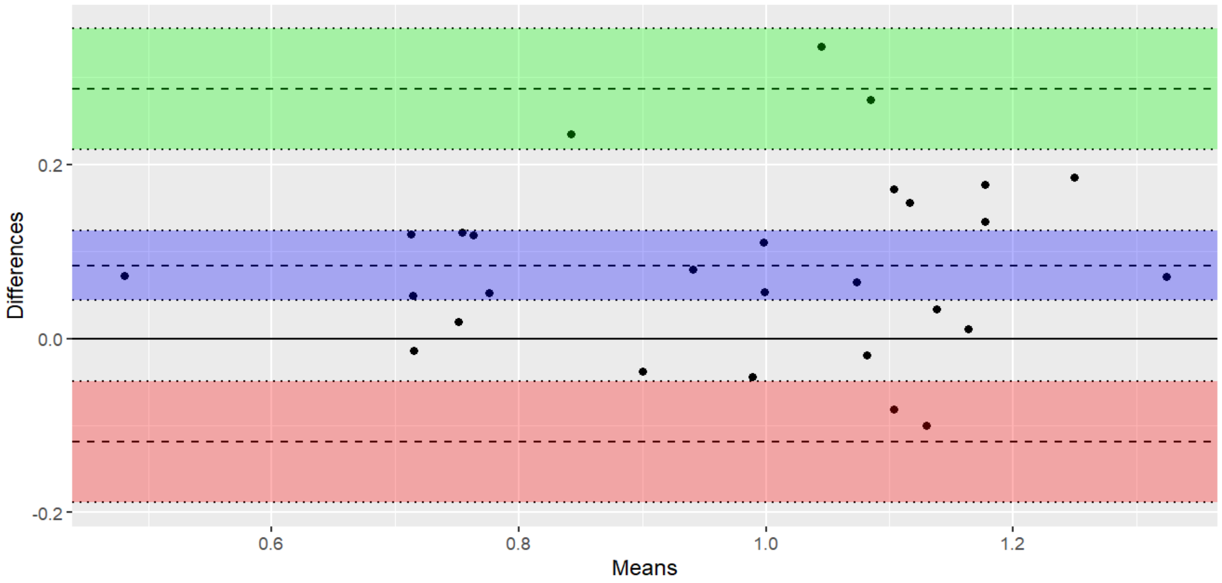

Chirp126 time 3 vs time 1 fast walk

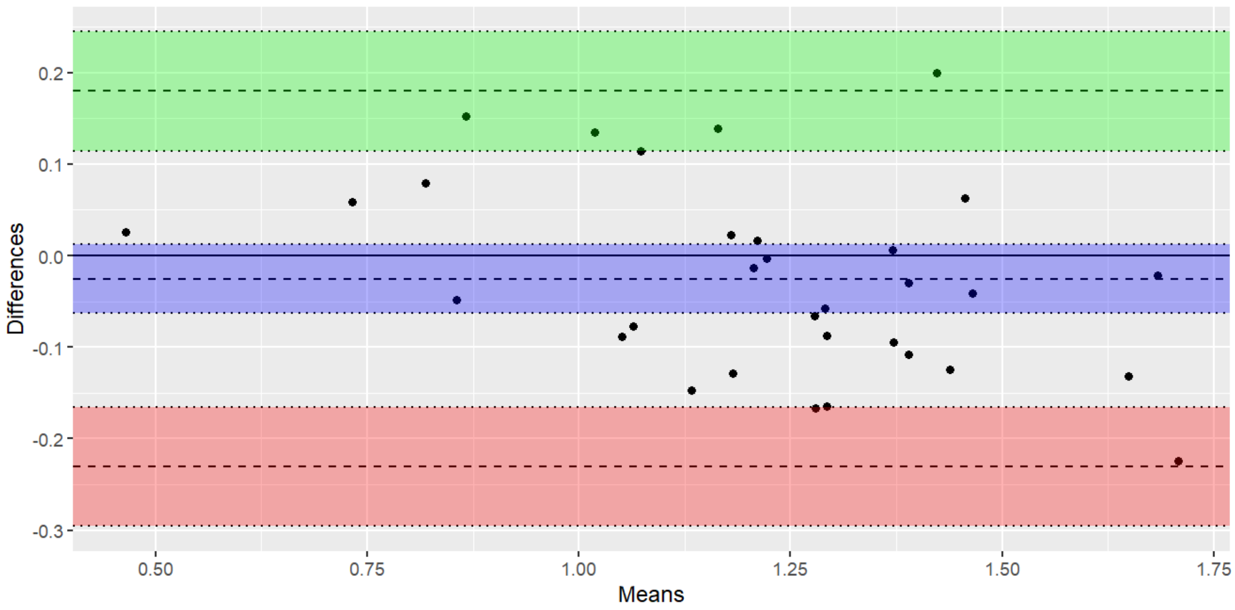

Chirp201 time 3 vs time 1 all experimental conditions

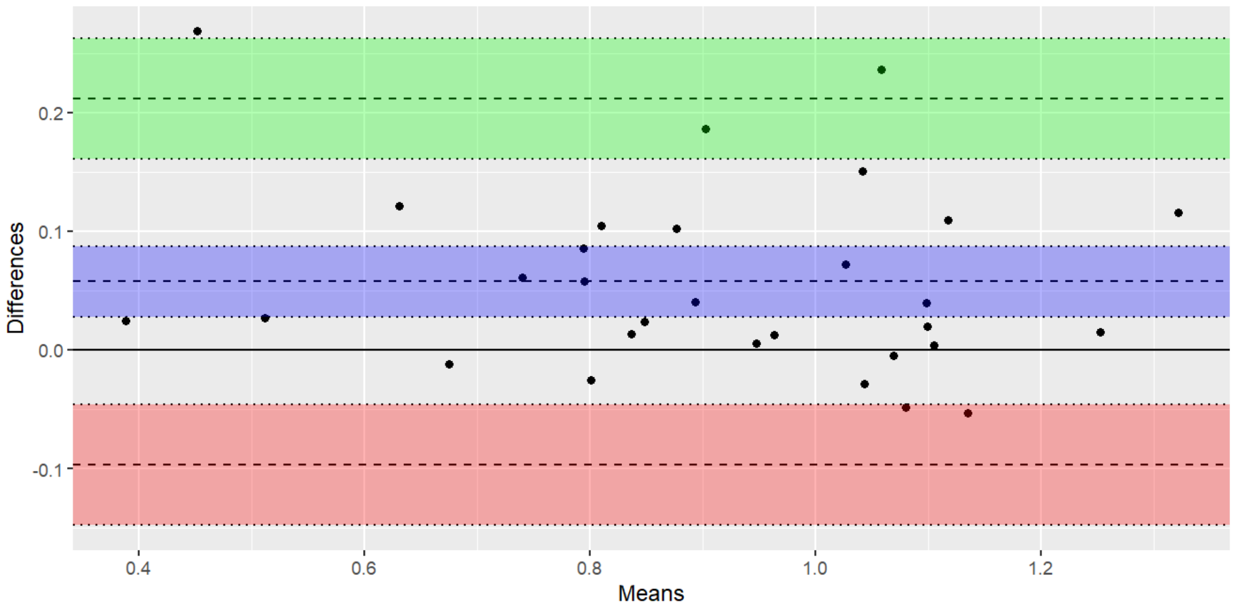

Chirp201 time 3 vs time 1 normal walk

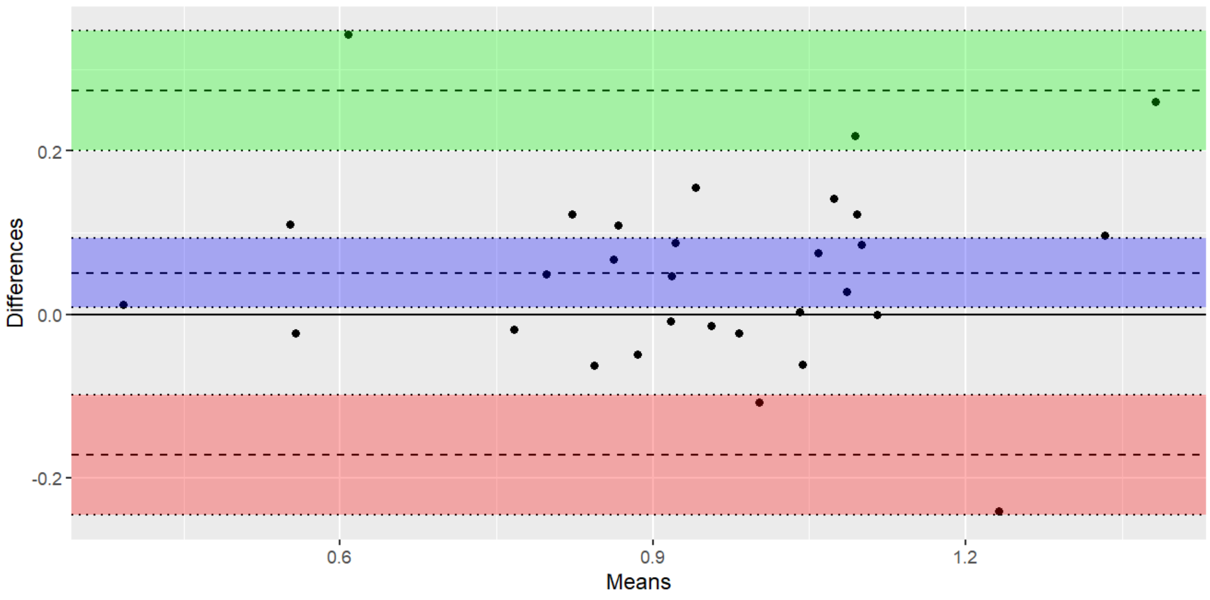

Chirp201 time 3 vs time 1 obstacle walk

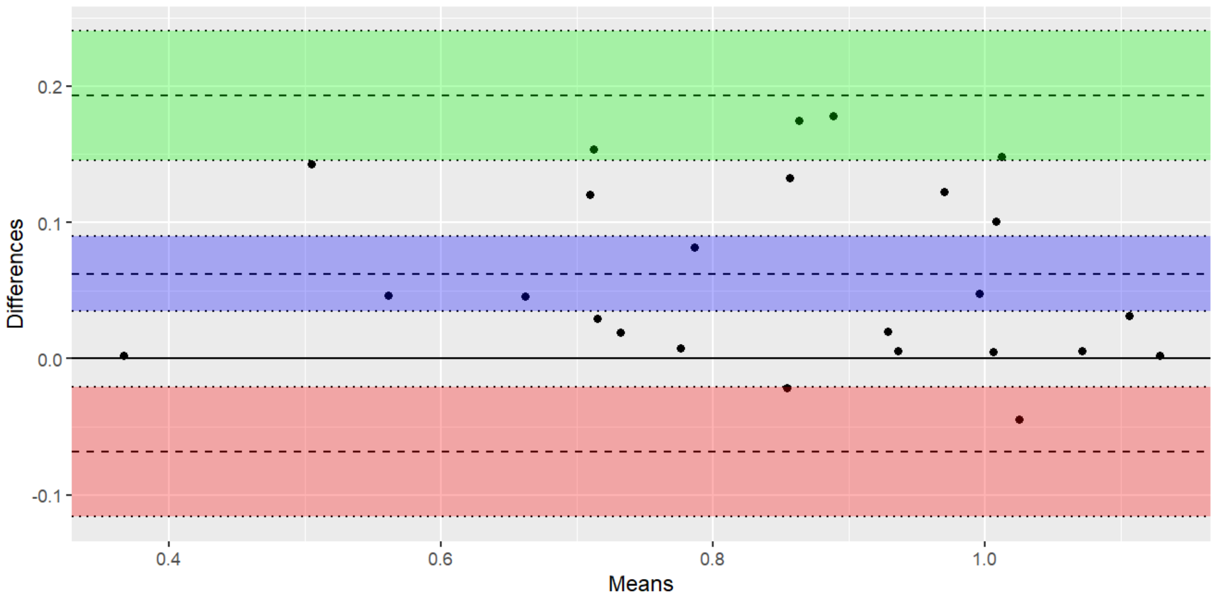

Chirp201 time 3 vs time 1 walk and talk

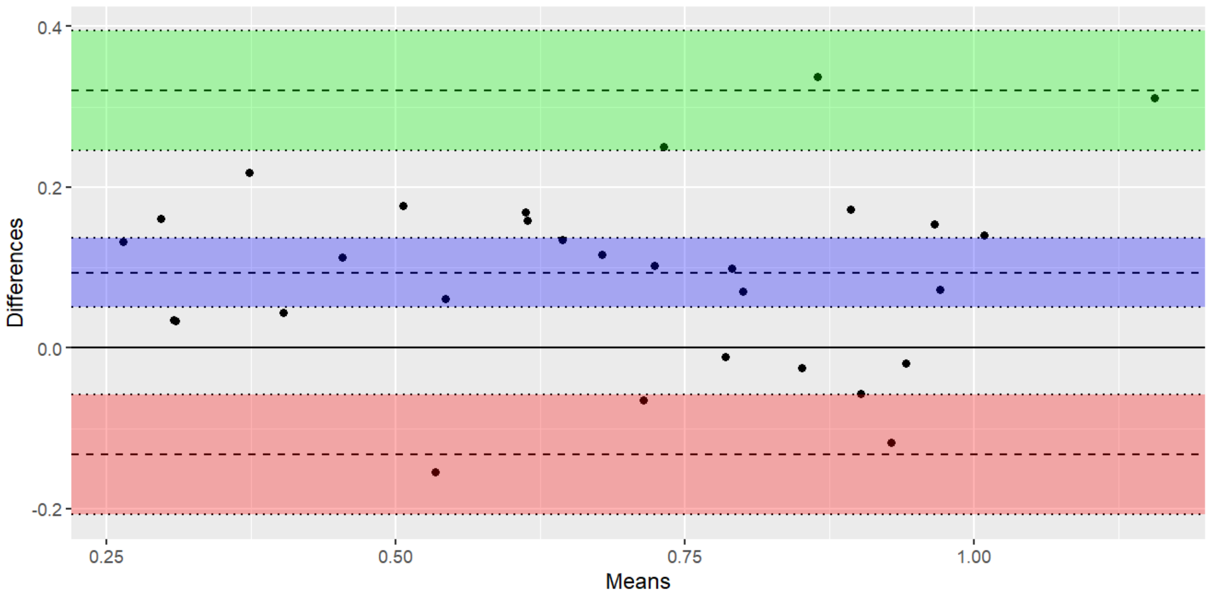

Chirp201 time 3 vs time 1 narrow walk

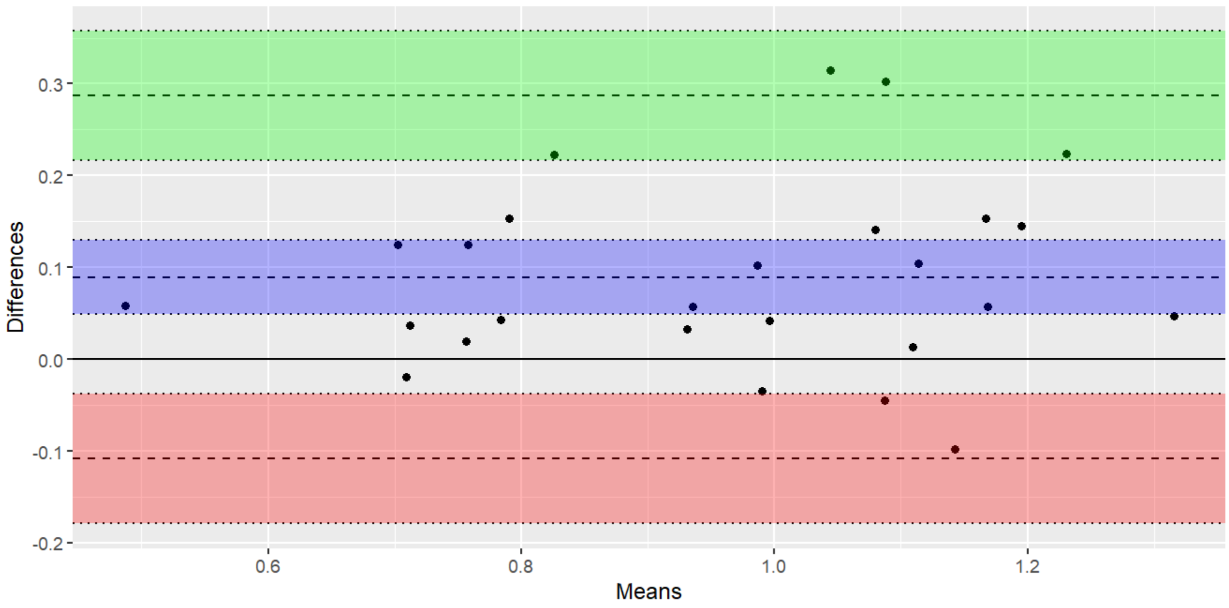

Chirp201 time 3 vs time 1 fast walk

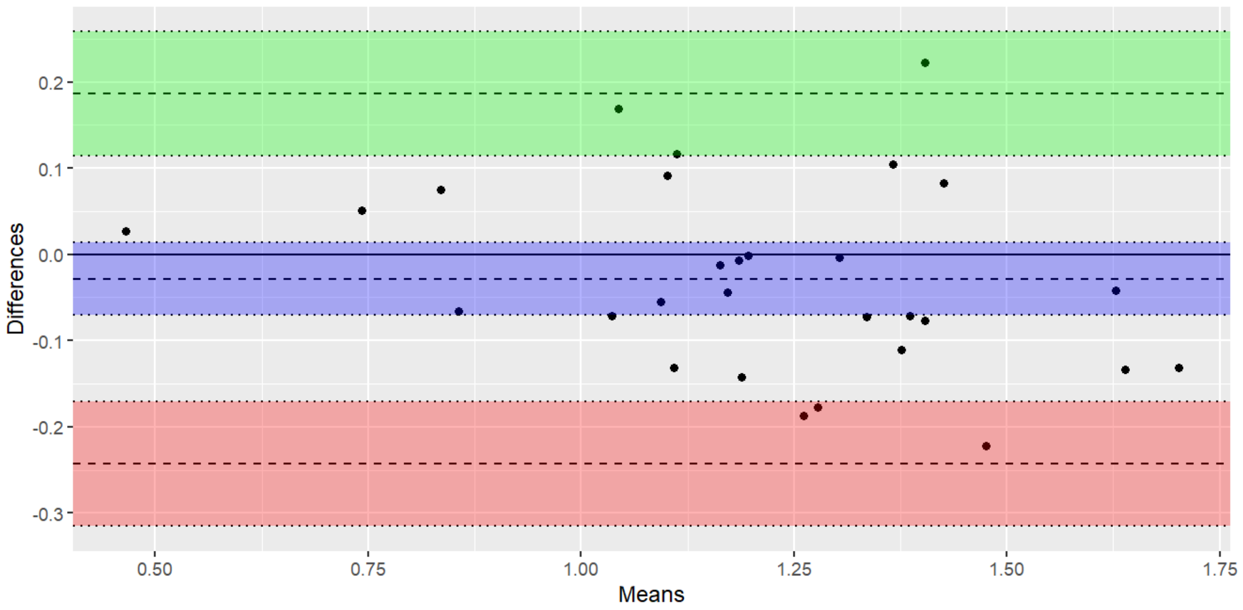

Supplement: S1 File — (PDF) [file pone.0307347.s002.pdf]
