# Supplementary material for: The reliability and validity of a non-wearable indoor positioning system to assess mobility in older adults: A cross-sectional study
Source: PLoS One. 2025 Apr 25;20(4):e0307347. doi: 10.1371/journal.pone.0307347 (PMC12027111; doi:10.1371/journal.pone.0307347)
Supplement: Attachment — Submitted filename: Response to reviewers and editor_Chirp manuscript.docx [file pone.0307347.s005.docx]

Thank you for reviewing our manuscript entitled “The reliability and validity of a non-wearable indoor positioning system to assess mobility in older adults: A cross-sectional study". We have addressed your comments below. The line and page numbers are associated with the tracked changes version of the manuscript.

Reviewer #1: The manuscript titled " The reliability and validity of a non-wearable indoor positioning system to assess mobility in older adults: A cross-sectional study" presents an interesting research topic that may benefit researchers and health care (measuring gait parameters in older adults). The authors have well-written elaborately well and were able to clearly communicate their findings. The authors also explained the content related to evaluating Chirp's inter-sensor reliability, intra-session test-retest reliability, and concurrent validity in a clinical setting. Moreover, the study involved 35 older adults with an average age of 75.5 years, most of whom were women living alone in an urban area. Participants walked under various experimental conditions, and their movements were recorded using the Chirp sensor and the ProtoKinetics Zeno™ Walkway. Including, the results show that Chirp demonstrated excellent reliability and validity across all conditions. However, the association between Chirp and cognitive scores was weak, and there was no significant association between Chirp and health-related quality of life. The findings suggest that Chirp is a reliable tool for monitoring older adults' mobility.

There are some areas that require modifications in order to justify grounds for publication within PLOS ONE. Please see below for my specific and generally requested modifications.

Specific Edits:

1. In the Abstract section, the authors evaluated the inter-sensor reliability, intra-session test-retest reliability, and concurrent validity of Chirp in a clinical setting. However, the term “walking speed” was not mentioned in this section.

We included the following sentence in the abstract: “*We assessed participants walking speed during normal and adaptive locomotion experimental conditions (walking-while-talking, obstacle, narrow walking, fast walking).*” (Page 2, Lines 36-37). We have also modified the following sentence in abstract: “*Chirp walking speed inter-sensor reliability ICC_(A,1)_=0.999[95% Confidence Interval [CI]: 0.997 to 0.999] and intrasession test-retest reliability [ICC_(A,1)_ =0.921, 95% CI: 0.725 to 0.969] were excellent across all experimental conditions. Chirp walking speed concurrent validity compared to the ProtoKinetics Zeno^TM^ Walkway was excellent across experimental conditions [ICC_(A,1)_= 0.993, 95% CI: 0.985 to 0.997].” (Page 2, Lines 44-49) and “We found a weak association between walking speed ~~Chirp~~ and cognition scores using the Montreal Cognitive Assessment across experimental conditions (estimated β-value= 7.79, 95% CI: 2.79 to 12.80) and no association between the walking speed ~~Chirp~~ and health-related quality of life using the 12-item Short Form Survey across experimental conditions (estimated β-value=6.12, 95% CI: -7.12 to 19.36)*” (Page 2, Line 49-53). We have additionally modified these results sentences throughout the paper.

2. In the Outcomes section, it is unclear whether the 4-meter ProtoKinetics Zeno™ Walkway and the 12-foot ProtoKinetics Zeno™ Walkway refer to the same measurement. The authors should clarify and ensure consistency in the units of measurement used.

We searched the document and changed “12-foot” to “4-meter” for consistency. The change was made on page 8, line 225 in the outcome section.

3. In the Data Collection section, the authors stated that “The study visit was divided into three blocks.” However, “Block 2” was not mentioned in the study and should be addressed.

Block 2 refers to the time when demographic data was collected. We have clarified this point with the following sentence: “*During “Block 2”, we collected demographic characteristics using the PROGRESS questionnaire [29], frailty scores with the Fit-Frailty Assessment and Management Application [30], cognitive scores using the MoCA [32], and health-related quality of life with the SF-12 questionnaire [31].” (Page 7, Lines 177-178).*

*4. In the Results section, the phrase “Of the 700 walks (35 participants × 2 blocks × 5 experimental conditions × 2 trials each)*” implies that there were 2 blocks. Is this correct?

There were three blocks, so we have rewritten the sentence to clarify this: “*Of the 700 walks ([35 participants] × [blocks 1 and 3] × [5 experimental conditions] x [2 trials each])*” (Page 9, line 246).

5. In Tables 2 and 3, the walking speed for each condition should be presented in both tables for consistency.

We included the gait speed for each condition in table 2 (see pages 10-11, line 262).

6. I suggest the authors include a Bland-Altman analysis (including a Bland-Altman plot) to assess the agreement in evaluating Chirp's inter-sensor reliability, intra-session test-retest reliability, and concurrent validity.

We included Bland Altman analysis and plot as a supplementary file (see S1 File) and made note of the file in the results section (“*The results of the Bland-Altman analysis can be found in S1 File. The estimated biases were very small; however, in some cases, the estimated biases were significant (significant values are highlighted in yellow in the S1 File). There were no patterns in the Bland-Altman plots*.” Page 12, Lines 277-280).

Reviewer #2:

1. Author should add more recent studies that can be related to mobility among older adults. need to compare to existing support system to mobility and what is current lack of mobility support system and how this system are much more better and give difference compare to existing system.

We included several studies on different types of devices that can be used to assess mobility in adults and older adults with comorbidities. Of the articles we cited, two are reviews (one scoping review and one literature review, citations 24 and 26). To address your point, we have included the following paragraph in the introduction: “*A recent scoping review of 95 reviews (51 systematic reviews and 44 reviews) identified several types of technologies that can be used for in-home rehabilitation [24]. The authors of the scoping review found that technologies such as virtual reality, augmented reality, and robotics should not be used independently for home-based rehabilitation due to usability and safety reasons [24]. Nevertheless, one major finding revealed technologies such as gamification, digital mobile apps, and Internet of Things can increase end-user engagement in the rehabilitation process as it allows for self-monitoring [24]. The biggest limitations to the current devices identified in this scoping review were the high costs to purchase the devices, the usability of the system (e.g., requires regular calibration to reduce errors), and the limited number of studies regarding the systems effectiveness [24–26]. Non-wearable, passive technologies with minimal set-up and calibration may be a more user-friendly solution for older adults [21,23].”*  (Page 4, lines 87-100).

2. is there any results related to acceptance from older adults, for example how older adults rate their comfort when they using this system, and perhaps can add features of this system that are practical and user-friendly the older adults.

We also conducted a feasibility and acceptability trial with the Chirp device. The results indicate that the Chirp device is a feasible and reliable artificial intelligence technology that can be used at home by community-dwelling older adults. We have referenced the following paper throughout the manuscript (see reference 28):

 “A Papaioannou, P Siva, G Ioannidis, IB Rodrigues, J Adachi, A Rabinovich, R Swance, S Tariq, A Kouroukis, C Leckie, A Lee. P Hewston. Chirp artificial intelligence at home. Poster presented at: Canadian Geriatrics Society 2024 Annual Scientific Meeting; Apr 25, 2024. Vancouver, BC.”

3. should include ethical considerations approval and how ethics play role when involving this group of respondents?

We have added the following sentences to address your point: “*The Chirp device is a privacy preserving device that will not collect or store personal identifying information or personal health information. The Chirp device was linked to a unique study identification number for each participant and participants cannot be identified using the Chirp device or smartphone app. For the purposes of this study, the Chirp device did not record or analyze audio, and the industry sponsor ensured these applications remained disabled for the duration of the study*.” (Page 6, lines 147-152)

4. should add more studies and research in this study

Thank you for your comment. We have addressed the issue under your first comment.

Journal requirements:   When submitting your revision, we need you to address these additional requirements.

 1. Please ensure that your manuscript meets PLOS ONE's style requirements, including those for file naming. The PLOS ONE style templates can be found at

https://journals.plos.org/plosone/s/file?id=wjVg/PLOSOne_formatting_sample_main_body.pdf and

https://journals.plos.org/plosone/s/file?id=ba62/PLOSOne_formatting_sample_title_authors_affiliations.pdf

 We have reviewed and confirm that the manuscript meets the PLOS ONE style requirements.

2. We noticed you have some minor occurrence of overlapping text with the following previous publication(s), which needs to be addressed:

 https://www.mdpi.com/1424-8220/24/4/1056

There is some overlap with the above paper as the Chirp study is part of a larger study. The MDPI paper is also part of the larger Chirp stud and utilized the same methods reported in our paper. We have attempted to reword some of the sentences, but there will be slight overlap as the methods are the same between both papers.

In your revision ensure you cite all your sources (including your own works), and quote or rephrase any duplicated text outside the methods section. Further consideration is dependent on these concerns being addressed.

 3. We note that the grant information you provided in the ‘Funding Information’ and ‘Financial Disclosure’ sections do not match. When you resubmit, please ensure that you provide the correct grant numbers for the awards you received for your study in the ‘Funding Information’ section.

 We have updated the Funding Information and Financial Disclosure section: “*We received funding from the Federal Economic Development Agency for Southern Ontario (FedDev), the Southern Ontario Pharmaceutical and Health Innovation Ecosystem (SOPHIE), and Chirp Inc. The funders had no role in study design, data collection and analysis, or decision to publish. The funder did play a role in the preparation of the manuscript*”.

4. Thank you for stating the following financial disclosure:

 [We received funding from the Federal Economic Development Agency for Southern Ontario (FedDev) -

Southern Ontario Pharmaceutical and Health Innovation Ecosystem (SOPHIE) and Chirp Inc].

Please state what role the funders took in the study.  If the funders had no role, please state: ""The funders had no role in study design, data collection and analysis, decision to publish, or preparation of the manuscript.""

If this statement is not correct you must amend it as needed.

Please include this amended Role of Funder statement in your cover letter; we will change the online submission form on your behalf.

 We have modified the statement to the following:  We have updated the Funding Information and Financial Disclosure section: “*We received funding from the Federal Economic Development Agency for Southern Ontario (FedDev), the Southern Ontario Pharmaceutical and Health Innovation Ecosystem (SOPHIE), and Chirp Inc. The funders had no role in study design, data collection and analysis, or decision to publish. The funder did play a role in the preparation of the manuscript*

5. Thank you for stating the following in the Competing Interests section: [One of the authors on this paper (PS) is a co-owner of Chirp. He reviewed and provided input on the final manuscript. ]. Please confirm that this does not alter your adherence to all PLOS ONE policies on sharing data and materials, by including the following statement: ""This does not alter our adherence to  PLOS ONE policies on sharing data and materials.” (as detailed online in our guide for authors http://journals.plos.org/plosone/s/competing-interests).  If there are restrictions on sharing of data and/or materials, please state these. Please note that we cannot proceed with consideration of your article until this information has been declared.

One of the authors on this paper (PS) is a co-owner of Chirp. He reviewed and provided input on the final manuscript. We confirm that this does not alter our adherence to  PLOS ONE policies on sharing data and materials.

6. Please review your reference list to ensure that it is complete and correct. If you have cited papers that have been retracted, please include the rationale for doing so in the manuscript text, or remove these references and replace them with relevant current references. Any changes to the reference list should be mentioned in the rebuttal letter that accompanies your revised manuscript. If you need to cite a retracted article, indicate the article’s retracted status in the References list and also include a citation and full reference for the retraction notice.

We have reviewed our reference list and confirm that it is complete and correct.
