# Supplementary material for: The reliability and validity of a non-wearable indoor positioning system to assess mobility in older adults: A cross-sectional study
Source: PLoS One. 2025 Apr 25;20(4):e0307347. doi: 10.1371/journal.pone.0307347 (PMC12027111; doi:10.1371/journal.pone.0307347)
Supplement: Attachment — Submitted filename: Response_to_reviewers_and_editor_Chirp_manuscript_auresp_2.docx [file pone.0307347.s006.docx]

Thank you for reviewing our manuscript entitled “The reliability and validity of a non-wearable indoor positioning system to assess mobility in older adults: A cross-sectional study". We have addressed your comments below. The line and page numbers are associated with the tracked changes version of the manuscript.

Reviewers' comments:

**Reviewer #2:** Abstract Enhancement:

Abstract:

1) Consider explicitly emphasizing the novel contributions of the study in the abstract. While the findings are robust, a clearer statement of the implications for clinical applications could enhance the abstract’s impact.

We have included the following statement in the abstract: “*Detecting early sign of walking speed decline can allow older adults to seek preventative rehabilitation. Currently, there is a lack of consensus on which assessments to use to assess walking speed and how to continuously monitor walking speed outside of the clinic. Chirp is a privacy-preserving radar sensor developed to continuously monitor older adults' safety and mobility without the need for cameras or wearable devices. Our study purpose was to evaluate the inter-sensor reliability, intrasession test-retest reliability, and concurrent validity of Chirp in a clinical setting*.” (Page 2, lines 29 to 35).

2) The use of "walking speed" could be further clarified to highlight its critical role in mobility assessment.

We have included the following statement: “*We selected walking speed as a measure as it is a good predictor of functional mobility but also is associated with physical and cognitive functioning in older adults*.” (Page 2, lines 41 to 43).

Introduction:

1) The introduction could benefit from a stronger emphasis on the gaps in current gait assessment technologies and how the Chirp sensor addresses these gaps.

We have modified the introduction to emphasis the need to conduct this study using Chirp. Our main changes are in paragraphs 2 and 3: “*Walking speed and step length can be used to identify older adults at high risk of falls [14,15]. In a clinical setting, healthcare providers use methods based on physical examination and functional tests such as the walking speed test, the Short Physical Performance Battery [16], and the Timed Up and Go Test [17,18] to identify quantitative aspects of walking speed [14]. These functional tests only provide a snapshot of the patient’s walking speed [14]. Additionally, such physical examinations and functional measures take a considerable amount of time to perform in clinic and may not always be feasible to implement under real-world conditions due to limitations in space and access to equipment [14]. Moreover, many first line clinics do not have access to physiotherapy and occupational therapy who evaluate other walking parameters that could predict fall risk including cadence, stride length, or swing phase time [14].*

*Performing continuous mobility assessments of walking speed may be one solution to help healthcare providers intervene sooner and reduce injuries related to falls and mobility impairments [19,20]. A means of continuously assessing mobility passively during normal activities of daily living allows for real-time mobility assessment and early identification of mobility declines and impairments [21]. Early identification of mobility declines can result in pre-emptive interventions such as physiotherapy or functional strength and balance training to aid or improve mobility [22]. Identifying new digital technologies in rehabilitation could help identify early declines in mobility as such technologies may be opportunities to monitor health status at home. Systems for at home walking speed analysis can be divided into three major groups: non-wearable systems, wearable systems, and a combination of non-wearable and wearable systems [23]. Each system can provide precise information on walking patterns, step speed, step length and width, and static and dynamic balance under controlled conditions [23]. While there are several precise systems, there are limitations including high costs to purchase the devices, the usability of the system (e.g., requires regular calibration to reduce errors), and the limited number of studies regarding the system’s effectiveness [24–26].* *Wearable systems such as smartphone apps are a popular method to collect data on gait parameters including gait speed and step length [27]. Although smartphone use is on the rise among older adults, its use is restricted by the need to always carry the smartphone and be connected to Wi-Fi. Other technologies such as virtual reality, augmented reality, and robotics should not be used independently for home-based rehabilitation due to usability and safety reasons [24]. Non-wearable, passive technologies with minimal set-up and calibration may be a more user-friendly solution for older adults [21,23].*

*Chirp (Waterloo, Ontario, Canada) is a privacy-preserving, non-wearable sensor that uses radar to passively collects walking parameters in older adults [20]. The radar-based approach in the Chirp technology can also be used to monitor walking parameters within the home and requires minimal set-up and calibration from the end-user [20]. Since radar technology is safe, easy to use, inexpensive, and inconspicuous while maintaining privacy, it has emerged as the best option for ongoing walking monitoring at home [28]. The Chirp sensor may be a realistic technology to assess mobility in clinical settings. Before the Chirp sensor can be used in clinic, the system’s psychometric properties should be tested against standard clinical mobility assessment metrics. This study evaluated the inter-sensor reliability, intrasession test-retest reliability, and concurrent validity of the Chirp sensor among older adults in a clinical setting. Our secondary objective was to determine if there is an association between walking speed using the Chirp sensor and self-reported mobility disability and cognition.*” (Pages 4 and 5 lines 83 to 122)

2) Including more recent and diverse studies in the literature review would strengthen the relevance of the research context.

We included several types of reviews to support the introduction; we limited our search to reviews from the last 5-years. Please see the following references:

23.	Argañarás JG, Wong YT, Begg R, Karmakar NC. State-of-the-art wearable sensors and possibilities for radar in fall prevention. Vol. 21, Sensors. MDPI; 2021.

24.	Arntz A, Weber F, Handgraaf M, Lällä K, Korniloff K, Murtonen KP, et al. Technologies in Home-Based Digital Rehabilitation: Scoping Review. JMIR Rehabil Assist Technol. JMIR Publications Inc.; 2023;10.

25.	Do Nascimento LMS, Bonfati LV, Freitas MLB, Mendes Junior JJA, Siqueira HV, Stevan SL. Sensors and systems for physical rehabilitation and health monitoring—a review. Vol. 20, Sensors (Switzerland). MDPI AG; 2020. p. 1–28.

26.	Cogollor JM, Rojo-Lacal J, Hermsdörfer J, Ferre M, Teresa Arredondo Waldmeyer M, Giachritsis C, et al. Evolution of cognitive rehabilitation after stroke from traditional techniques to smart and personalized home-based information and communication technology systems: Literature review. Vol. 20, JMIR Rehabilitation and Assistive Technologies. 2018.

27.	Brognara L. Gait Assessment Using Smartphone Applications in Older Adults: A Scoping Review. Vol. 9, Geriatrics (Switzerland). Multidisciplinary Digital Publishing Institute (MDPI); 2024.

28.	Zeng X, Báruson HSL, Sundvall A. Walking Step Monitoring with a Millimeter-Wave Radar in Real-Life Environment for Disease and Fall Prevention for the Elderly. Sensors. MDPI; 2022 Dec 1;22(24).

Discussion and Implications:

1) Strengthen the discussion around how this technology could be integrated into existing clinical workflows and what barriers might exist for widespread adoption.

We included the following paragraph to discuss how the technology can be integrated into clinical workflow; however, we will need several studies to troubleshoot the ethical implications of implementing such technology in hospitals and clinical practice: “*Chirp sensors are an in-home device that can simply be mounted without the need to modify the walls. Additionally, this system has sensors that monitor activity without cameras or the need to wear equipment or push a button. This passive method of collecting data may be integrated into clinical practice to aid healthcare professional when making medical decisions. The results of the Chirp sensor can be used in clinical practice in two ways. One, healthcare professionals could access reports through the Chirp system that summarize important gait parameters, although, accessing such data may be time consuming for healthcare professionals who have already establish a routine. Alternatively, introducing radar into clinics may be more practical [45]. Careful consideration should be given to ethical and privacy protection when using radar in clinical practice such as how patient data is stored, training health personnel to establish standardized operational procedures, and ensuring system compatibility with other medical devices [45]. One of the primary ethical concerns associated with using any type of radar system in hospitals and clinical practice is patient privacy [45]. Hospitals will need to develop clear policies and procedures to obtain consent. Consent may involve explaining the purpose of the radar system, the potential risks of technology, and how the data will be collected, used, stored, shared and destroyed [45]. There are still several barriers that need to be considered before radar systems can be full adopted and used in hospital and clinical settings.*” (Page 16, lines 361 to 377).

2) Discuss the potential for real-world application in multi-user settings, as highlighted briefly, but elaborate on the challenges and next steps for such scenarios.

Although the discussion regarding multiple walking individuals is beyond the scope of this paper, we have included the following paragraph to address your point as it is important to mention our next steps: “*The next steps may be to evaluate the Chirp sensors in situations where multiple individuals are walking to increase its applicability to real world scenarios. The possibility of the coexistence of multiple walking individuals is high, especially in the outpatient setting where the hallways are shared with several individuals including patients and their caregivers, hospital staff, and healthcare providers. Future studies will need to address the problem of tracking and monitoring multiple walking individuals in the same environment using advanced mathematical algorithms that can remove multipath effects or ghosts; ghosts occur when a transmission signal is bounced of another object [44]. Such algorithms may focus on identifying and separating reflected signals that may occur when two or more individuals walk closely together. In contrast to most other radar systems that only acquired walking speed of a single individual, utilizing multipath effect algorithm methods enables the simultaneous extraction of walking speed from numerous closely spaced participants [44]. Future test with Chirp sensors will need to understand how radar ghost due to multipath return can be mitigated*.” (Page 15 and 16, lines 348 to 360).
